# Benign Bronchial Tumours: A Rare Case of Endobronchial Fibroma With Hilar–Mediastinal Lymphadenopathy

**DOI:** 10.1155/crpu/8730962

**Published:** 2026-06-02

**Authors:** Giuseppina Marrazzo, Teresa Ferrazzo, Giuseppe Failla, Francesco Giuseppe Tropea, Nicola Montenegro, Gianluca Ippolito, Fabio Alfredo Nania, Carlo Gentile, Annamaria Lavecchia, Corrado Pelaia, Girolamo Pelaia

**Affiliations:** ^1^ Respiratory Unit, A.O.U. “Renato Dulbecco”, Catanzaro, Italy; ^2^ Department of Health Sciences, University “Magna Græcia”, Catanzaro, Italy, unicz.it; ^3^ Diagnostic and Therapeutic Bronchoscopy Unit, ARNAS “Civico e Benfratelli”, Palermo, Italy; ^4^ Anesthesia and Intensive Care Unit, “Santa Maria Goretti” Hospital, Latina, Italy; ^5^ Pathology Unit, A.O.U. “Renato Dulbecco”, Catanzaro, Italy; ^6^ Department of Medical and Surgical Sciences, University “Magna Græcia”, Catanzaro, Italy, unicz.it

**Keywords:** benign bronchial tumour, EBUS-TBNA, endobronchial fibroma, laser resection, lymphadenopathy, rigid bronchoscopy

## Abstract

**Background:**

Endobronchial fibroma is a rare benign tumour that can mimic lung cancer, especially when accompanied by airway obstruction and hilar–mediastinal lymphadenopathy.

**Case Presentation:**

A 73‐year‐old man under radiological surveillance for pulmonary nodules developed interval growth of a middle‐lobe nodule with new hilar–mediastinal lymphadenopathy and an obstructing B4 endobronchial mass. Bronchoscopy revealed a smooth, firm, rubbery polypoid lesion. Tissue sampling included fine‐needle aspiration of the endobronchial mass and EBUS‐TBNA of nodal stations (7, 11Ri and 11 L). Histology showed fibromatous tissue without atypia; lymph nodes exhibited inflammatory changes. The lesion was fully removed via rigid bronchoscopy with forceps and laser, leading to symptom relief and planned radiological follow‐up. We propose a stepwise evaluation pathway for endobronchial tumours when lymphadenopathy is present, highlight pathology features that distinguish fibroma from malignant and benign mimickers and outline practical considerations for bronchoscopic resection.

**Conclusion:**

In patients with concurrent endobronchial mass and lymphadenopathy, a targeted approach using EBUS‐TBNA can exclude nodal metastasis and support definitive, minimally invasive treatment, potentially avoiding unnecessary surgery.

## 1. Introduction

Fibroma is a rare benign bronchopulmonary tumour that primarily occurs in the lung parenchyma or pleura [1, 2]. It is often discovered on routine chest radiography, and histopathological examination provides the definitive diagnosis. Endobronchial fibroma is extremely uncommon, with only a few cases reported [3]. Fibromas may be asymptomatic or may cause obstructive pneumonia or atelectasis [4]. A bronchial fibroma can be small and amenable to bronchoscopic removal using forceps or laser [5], or it can be large, requiring lobectomy due to bronchial occlusion [6]. The aetiology of fibromas remains unknown; however, they generally have a favourable prognosis. Because clinical and radiological features can overlap with those of malignant disease—especially when lymphadenopathy is present—these lesions pose diagnostic and management challenges.

We report a rare endobronchial fibroma in a patient with a family history of malignancy and pulmonary nodules under follow‐up. We emphasise three practical contributions: (i) a step‐by‐step diagnostic approach for suspected benign endobronchial tumours in high‐risk patients; (ii) key pathology pointers to differentiate fibroma from malignant mimics and other benign fibrous proliferations; and (iii) pragmatic guidance for optimising bronchoscopic therapy and follow‐up. These points are aimed at improving clarity, clinical usefulness and the value of our report within the existing literature.

## 2. Case Report

**Figure 1:**
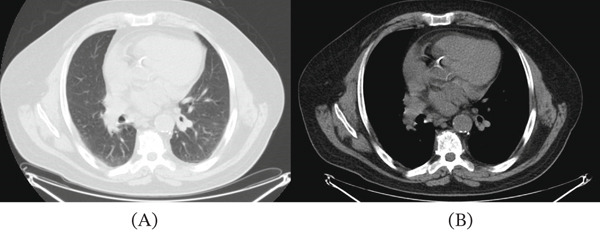
Chest computed tomography (CT). (A) Axial image in lung window showing the hilar region and (B) axial image in mediastinal window demonstrating hilar–mediastinal structures.

A 73‐year‐old man was undergoing follow‐up for pulmonary nodules in the posterior segment of the left upper lobe, initially identified on chest‐computed tomography (CT) performed for dyspnoea. At subsequent radiographic review approximately 3 months later, there was an increase in the size of one nodule (maximum diameter 24 × 18 mm, previously 14 × 9 mm), enlargement of hilar–mediastinal lymph nodes (maximum diameter 24 mm, previously 17 mm), and parenchymal thickening in the lateral segment of the middle lobe (Figure 1).

For diagnostic clarification, he was admitted to the pneumology unit for endobronchial ultrasound‐guided transbronchial needle aspiration (EBUS‐TBNA) [7]. His history included a family history of lung cancer and tobacco exposure (former smoker for approximately 30 years (~30 cigarettes/day)). Comorbidities comprised hypertension, ischaemic heart disease, chronic obstructive pulmonary disease (COPD), hepatic steatosis, chronic renal failure, benign prostatic hypertrophy, diabetes mellitus and dyslipidaemia, all under pharmacological treatment.

During hospitalisation, pulmonary function tests (PFTs) while on inhaled corticosteroid (ICS)/long‐acting *β*2‐agonist (LABA)/long‐acting muscarinic antagonist (LAMA) therapy documented a mild restrictive ventilatory defect. Diffusing capacity of the lung for carbon monoxide (DLCO) measured by the single‐breath method was normal. The 6‐min walk test showed no exertional desaturation [8]. Overnight cardiorespiratory monitoring revealed chronic latent respiratory failure secondary to moderate obstructive sleep apnoea syndrome (OSAS), with an indication for continuous positive airway pressure (CPAP). Arterial blood gases and blood tests were within normal ranges. Echocardiography showed eccentric left‐ventricular hypertrophy, preserved biventricular systolic function, mild–moderate mitral regurgitation, and Grade II diastolic dysfunction.

**Figure 2:**
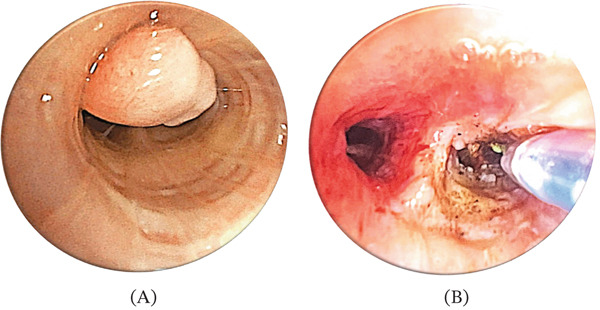
Bronchoscopic view of the right middle lobe B4 segment showing a smooth, sessile, hard‐elastic endobronchial mass partially occluding the (A) lumen (pre‐resection) and (B) during its removal by laser technique.

Flexible bronchoscopy demonstrated, in the right bronchial tree, a lesion in the lateral segmental branch (B4) of the middle lobar bronchus: a smooth‐surfaced, firm, rubbery polypoid mass with a broad base along the anterolateral wall, suspicious for a neoplasm. Fine‐needle aspiration (FNA) of the lesion was performed using a 21‐gauge needle for histologic and cytologic examination. Given the radiologic and endoscopic evidence of lymphadenopathy, EBUS‐TBNA targeted the subcarinal (Station 7), right interlobar inferior (11Ri), and left interlobar (11 L) nodal stations using a 21‐gauge needle (Olympus BF‐UC190F). With an indication for laser‐assisted removal, the lesion was completely resected by rigid bronchoscopy at a Level II interventional pulmonology centre. Rigid bronchoscopy confirmed a mass suboccluding the B4 segmental branches, which was removed with forceps and laser. The lesion exhibited a smooth surface with a broad fibrous implantation base (Figure 2).

**Figure 3:**
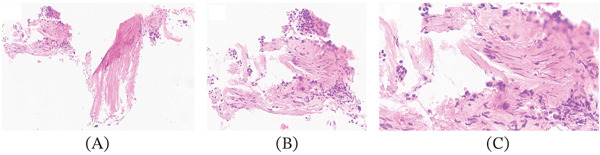
Haematoxylin and eosin‐stained endobronchial biopsy, original magnification (A) ×25, (B) ×100, (C) ×200: mesenchymal, fibrous neoplasm composed of cells with spindle nuclei and eosinophilic cytoplasm blending with surrounding stroma and bronchioloalveolar sloughing cells.

The histological examination, using haematoxylin (Shanghai Chemical Reagent Co. Ltd., Shanghai, China) and eosin (Tianjin Bodi Chemical Co. Ltd., Tianjin, China) stains, performed on the biopsy with the FNA technique and on the surgical specimen, showed fibromatous tissue with cellular elements from the bronchial lining, without significant atypia (Figure 3). Histology of the lymph node sample revealed the presence of inflammatory material.

The left upper‐lobe nodular lesion, which initially prompted radiological follow‐up, was re‐evaluated with CT during follow‐up and was reported as radiologically stable, with no significant changes in size or morphology. However, follow‐up imaging was not available for inclusion in this report. The patient was scheduled for continued radiological surveillance according to standard clinical practise.

## 3. Discussion

Neoplasms of the tracheobronchial tree are uncommon and predominantly malignant [9]. In this case, a high pretest probability of cancer was suggested by the patient′s age, smoking history, pulmonary nodules and interval development of hilar–mediastinal lymphadenopathy. Bronchoscopy identified a smooth, firm endobronchial mass; lesion‐directed FNA and systematic nodal sampling with EBUS‐TBNA were pivotal in establishing the diagnosis. Concordant benign histology from both the airway lesion and mediastinal/hilar nodes supported a minimally invasive bronchoscopic resection.

Endobronchial fibroma is a rare benign tumour that can nevertheless cause clinically significant airway obstruction and related complications. Symptoms, when present, include cough, dyspnoea, and haemoptysis; obstructive pneumonia or atelectasis may occur [4, 10]. Important differential diagnoses include endobronchial hamartoma, inflammatory myofibroblastic tumour, fibroepithelial polyp and low‐grade sarcomatoid malignancies. Typical pathological features of fibroma include a hypocellular, heavily collagenised stroma with bland spindle cells, absence of necrosis, and rare mitotic figures. Although the aetiopathogenesis remains uncertain, fibromas have been linked to aberrant reparative processes following inflammation or injury [11].

Therapeutic strategy should be individualised according to lesion size, morphology, and degree of obstruction. For small sessile or pedunculated lesions, bronchoscopic resection—mechanical debulking with adjunctive laser or argon plasma coagulation—is an effective first‐line option, reserving anatomical resection for bulky, recurrent, or unresectable disease [5, 6]. In our patient, complete removal by rigid bronchoscopy with forceps and laser relieved obstruction and provided definitive treatment. Given the benign nature and complete endoscopic excision, noninvasive surveillance with periodic imaging and symptom monitoring is appropriate.

We also outline a practical assessment pathway for suspected benign endobronchial tumours presenting with lymphadenopathy: [1] bronchoscopic inspection and lesion‐directed sampling; [2] nodal evaluation by EBUS‐TBNA when lymphadenopathy is present; and [3] definitive bronchoscopic therapy when feasible. Providing succinct pathology descriptors may improve recognition and reporting by both clinicians and pathologists.

The reported stability of the left upper‐lobe lesion on follow‐up imaging further supports a nonaggressive clinical course. However, a limitation of this report is the unavailability of follow‐up CT images, which precludes visual documentation of this finding.

## 4. Conclusion

This case highlights the diagnostic challenge posed by the coexistence of an endobronchial mass and mediastinal lymphadenopathy in a high‐risk patient. A minimally invasive approach combining bronchoscopy and EBUS‐TBNA enabled accurate differentiation between benign and malignant disease. Bronchoscopic resection was effective in this selected case. However, conclusions should be interpreted cautiously given the single‐case nature, and further studies are needed to validate this diagnostic approach.

## Author Contributions

Giuseppina Marrazzo and Teresa Ferrazzo contributed equally to this study.

## Funding

No funding was received for this manuscript.

## Disclosure

An earlier version of this case report was presented at the XXIII National Congress of the Italian Society of Thoracic Endoscopy (SIET) in Florence from 17 to 19 October 2024.

## Ethics Statement

This study was conducted in accordance with the Declaration of Helsinki. According to institutional policies and national regulations, formal approval from the Ethics Committee was not required for the publication of a single case report. Written informed consent was obtained from the patient for the publication of clinical data and accompanying images.

## Conflicts of Interest

The authors declare no conflicts of interest.

## Data Availability

The data that support the findings of this study are available from the corresponding author upon reasonable request.
